# A Comparison of Pain Before and After Transfusion in Adult transfusion-dependent thalassemia (TDT) Using BPI-SF

**DOI:** 10.12688/f1000research.75952.1

**Published:** 2023-10-31

**Authors:** Uni Gamayani, Shenny Dianathasari Santoso, Asep Nugraha Hermawan, Pandji Irani Fianza, Ramdan Panigoro

**Affiliations:** 1 Department of Neurology, Faculty of Medicine, Universitas Padjadjaran/Dr. Hasan Sadikin Hospital, Bandung, 40161, Indonesia; 2 Department of Internal Medicine, Faculty of Medicine, Universitas Padjadjaran/Dr. Hasan Sadikin Hospital, Bandung, 40161, Indonesia; 3 Centre for Genetic Studies, Faculty of Medicine, Universitas Padjadjaran, Bandung, 40161, Indonesia

**Keywords:** Thalassemia; hemoglobin; pain; transfusion; BPI-SF; quality of life; questionnaire; pain assessment; adults.

## Abstract

Background

*Pain* is a complication in patients with transfusion-dependent thalassemia (TDT). There are several mechanisms underlying pain in people with thalassemia and low hemoglobin at the end of the transfusion cycle was the most etiology. Pain can develop into chronic pain and interfere with the quality of life. The Brief Pain Inventory Short Form (BPI-SF) can help identify pain in people with TDT. The present study aimed to compare pain before and after transfusion in adult TDT patients.

Methods

It was an analytical observational study using a cross-sectional design on adult TDT patients with pain who came to the Haemato-Oncology Clinic of Dr. Hasan Sadikin Hospital Bandung. This study was conducted from December 2020 to July 2021. All subjects were assisted in filling out the Indonesian version of the BPI-SF questionnaire hemoglobin levels were examined and before and after transfusion, then paired test analysis was performed using the Wilcoxon Test.

Results

This study is conducted on 60 adult TDT patients with symptoms of pain. The median value of pain intensity and pain interferes with life obtained from the Indonesian version of the BPI-SF score after transfusion decreased significantly compared to before transfusion (NRS 5 vs. 0 and 2.8 vs. 0; p=0.0001).

Conclusion

There is a significant difference in pain intensity and pain interfere with life in adults with TDT before and after transfusion. It is necessary to carry out pain assessments for thalassemia patients.

## Introduction

*Pain* is an unpleasant sensory and emotional experience for everyone but is not as a chief complaint and ignored in daily examinations. Recent studies have shown increasing pain reports in patients with thalassemia. ^1^^,^^2^ Thalassemia is the blood disorder occuring due to decreased or lost synthesis of one or more globin chains. ^3^ This disease is hereditary autosomal recessive due to mutations in the globin-forming gene. ^4^^,^^5^ The spectrum of thalassemia syndromes is based on the requirements for blood transfusions for survival, including transfusion-dependent thalassemia (TDT) and non-transfusion-dependent thalassemia (NTDT). ^6^

The World Health Organization or WHO states that about 7% of the world's population has the thalassemia gene, the highest incidence of up to 40% cases in Asia. β-thalassemia is found in Mediterranean countries, Central Asia, India, Southern China, North Africa and South America. Indonesia is included in the world's thalassemia belt area with the carriers incidence of thalassemia traits ranging from 6-10%. ^7^ There were more than 10.531 thalassemia patients in Indonesia according to the data from the Ministry of Health of the Republic of Indonesia in 2019. Dr. Hasan Sadikin Hospital Bandung is a referral hospital for West Java Province with the highest thalassemia cases in Indonesia reaching 40 percent of the national figure. ^8^

There are several mechanisms underlying pain in people with thalassemia. Low hemoglobin at the end of the transfusion cycle can cause pain in 45% cases and decrease after transfusion. ^9^ Low hemoglobin in thalassemia causes hypoxia and releases reactive oxygen species (ROS) through inhibition activity of oxygen-sensitive prolyl hydroxylases (PHDs), then causes activation of transient receptor potential ankyrin 1 (TRPA1) and transient receptor potential vanilloid 1 (TRPV1). ^10^^,^^11^ This protein activation will release substance P which plays a role in pain sensitization. ^12^ Transfusion can improve anemia, suppress erythropoiesis, and inhibit iron absorption in the gastrointestinal tract. Maintaining hemoglobin levels above 10 g/dL can prevent hyperplasia and bone marrow expansion, thereby preventing the development of skeletal abnormalities. ^4^^,^^13^

The pain effect in daily activities needs to be assessed using a pain screening tool. The Brief Pain Inventory (BPI) is the recommended pain screening tool to assess pain intensity and pain interfering with life. ^14^ The advantage of this questionnaire is available in a short form and easier to apply. This questionnaire can evaluate pain treatment and the pain impact on the patient's life quality with the sensitivity of 79.4%. ^15^^–^^18^

Pain in thalassemia needs to be identified because it can develop into chronic pain which can interfere the patients' life quality. Pain management in thalassemia depends on the pain assessment accuracy. This study aims to compare pain before and after transfusion in adult TDT patients.

## Methods

### Sample size

This study inclusion criteria was ≥18-year-old patients diagnosed with β-thalassemia with pain who underwent routine transfusion at the Hemato-Oncology Internal Medicine Clinic in Dr. Hasan Sadikin Hospital Bandung and was willing to participate in the research. Transfusion is initiated if the thalassemia diagnosis is confirmed based on severe anemia (hemoglobin level <7 g/dL for more than two weeks, excluding other anemia causes or hemoglobin level >7 g/dL with a facial deformity, impaired growth, presence of bone marrow expansion, and hepatosplenomegaly). The target for hemoglobin after transfusion is 13-14 g/dL; therefore, the transfusion frequency is every 2-4 weeks to achieve the hemoglobin target. The patient received 1-2 units of blood transfusion depending on the hemoglobin level before transfusion. The procedure was done in 3-4 hours. This adult selection as the subject (It ranges from the age of 18 in our hospital) was done since they were expected to be able to express pain more. Exclusion criteria was patients who had confirmed pain due to fractures/other diseases and had been diagnosed with anemia due to other causes.

The sample size of this study consists of subjects testing the questionnaire validity and the sample size for paired test analysis. The sample size for questionnaire validation in this study was determined using a correlation analysis formula obtained from 30 subjects. Subjects filling out the questionnaire complained various types of pain such as low back pain, nerve entrapment pain, radiculopathy, joint pain, and facial pain. The minimum sample size for paired test analysis is 46 research subjects. If 10% is added to anticipate data loss, the minimum sample size then is 51 research subjects. A total of 60 TDT patients were obtained according to the period of our research.

The written informed consent for this research was obtained from the patient and research ethics approval was obtained from the ethics committee of Padjadjaran University Bandung under the ethical clearance number of 198/UN6.KEP/EC/2021.

### Statistical analysis

**Figure 1.**
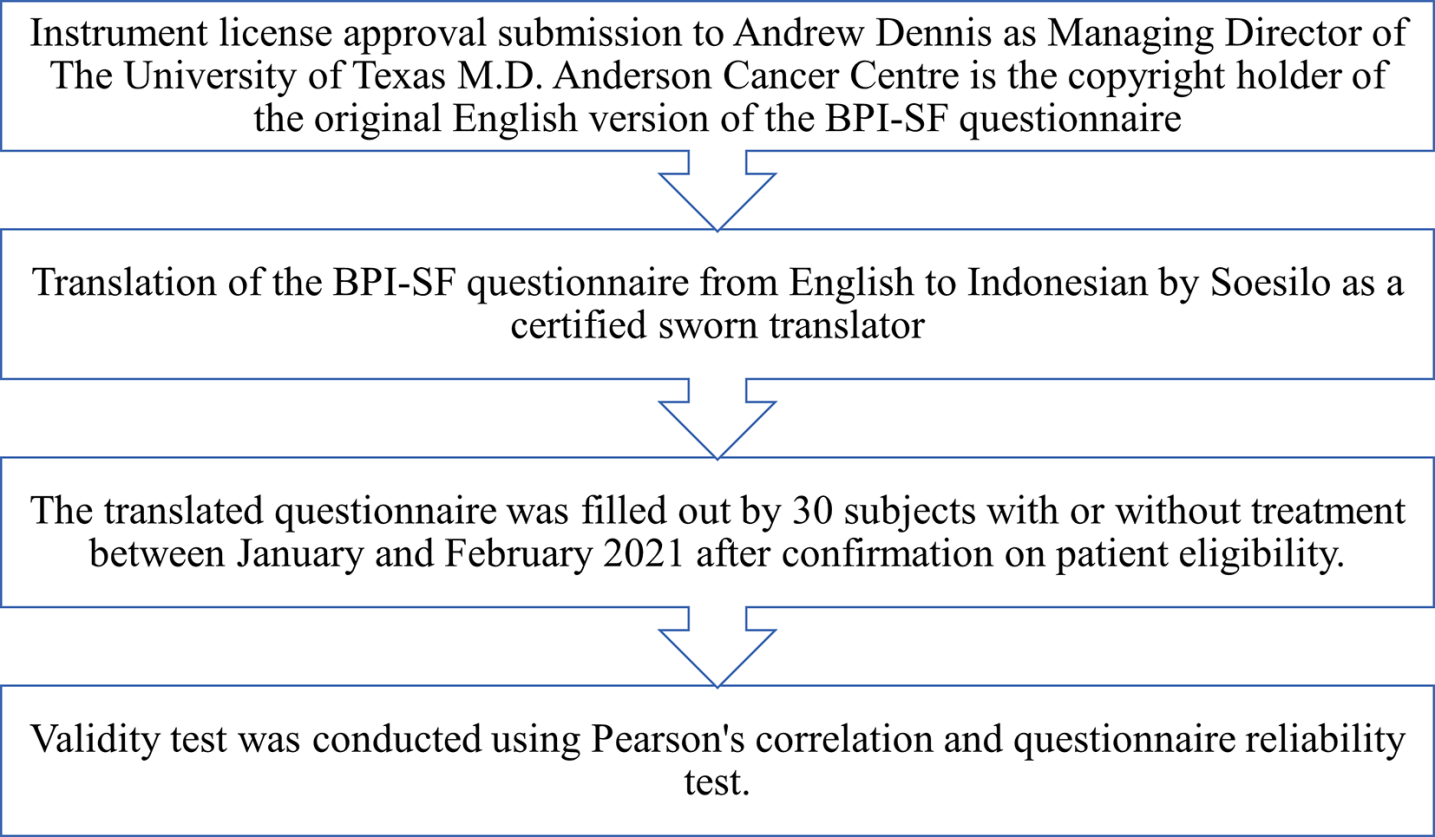
Flow of questionnaire vaildation.

This study was an analytical observation using a cross-sectional design conducted from December 2020 to June 2021. We perform the BPI-SF questionnaire validity test in the Indonesian version. The validity test stages were done according to the questionnaire validation guidelines using the following steps as described in Figure 1. ^19^

Sixty subjects meeting the inclusion criteria were explained about the study procedure and asked to sign an informed consent form if they were willing to participate; furthermore, demographic data collection and pain screening were carried out on the subject using the BPI-SF questionnaire. Patients were assisted before the transfusion and a maximum of one day after the transfusion in filling out the BPI-SF questionnaire in Indonesian version. The data was obtained by telephone interview due to the COVID-19 pandemic and the distant patient residence from the research site. Hemoglobin levels were examined before and after transfusion, and then a comparison test was performed with the Wilcoxon Test.

## Results

The validity test of this study uses Pearson's correlation. The results were obtained and processed using SPSS 24.0 software with a significance level (α)=0.05 (5%). The questionnaire is considered valid if it is greater than the Pearson product-moment correlation coefficient. The table correlation number (r table) is 0.30 with the number of samples (n)=30 people. All question instruments on the questionnaire variable are valid because the r-count is > 0.3. Reliability testing is carried out with internal consistency or the answers accuracy degree using *Cronbach's Alpha.* A measuring instrument is said to be reliable if the coefficient value of r is 0.7. All questionnaire variable instruments are reliable because of the r > 0.7 value.

This study was conducted to 60 people with thalassemia meeting the inclusion criteria and not included in the exclusion criteria. The subjects mean age was young adults, 26.1±9.1 years, and most of them were female (65%). The duration of being diagnosed with thalassemia ranged from 3-50 years. The subjects demographic distribution is described in Table 1.

**Table 1.** Demographic characteristics of research subjects.

Characteristics	N (%)
Age (years)	
Mean±SD	26.1±9.1
Gender	
Male	21(35)
Female	39(65)
Education	
Elementary school	7(11.7)
Junior high school	14(23.3)
Senior high school/Vocational school	32(53.3)
College	7(11.7)
Occupation	
Unemployed	49(81.7)
Employed	11(18.3)

### Pain characteristics of research subjects

**Figure 2.**
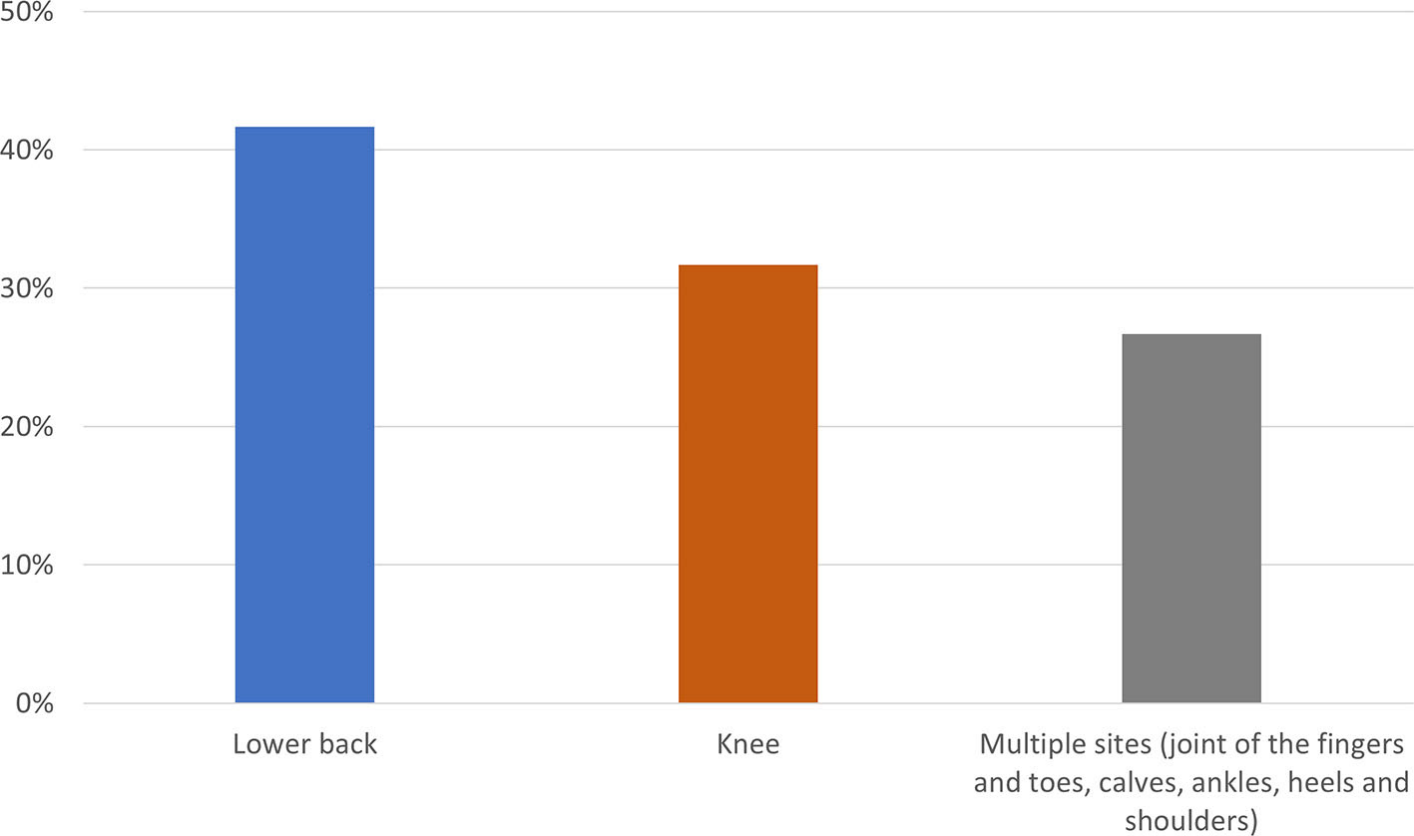
Description of pain location.

The pain location is experienced mainly in the lower back followed by the knee while the others complained it in more than one location, as seen in Figure 2.

**Figure 3.**
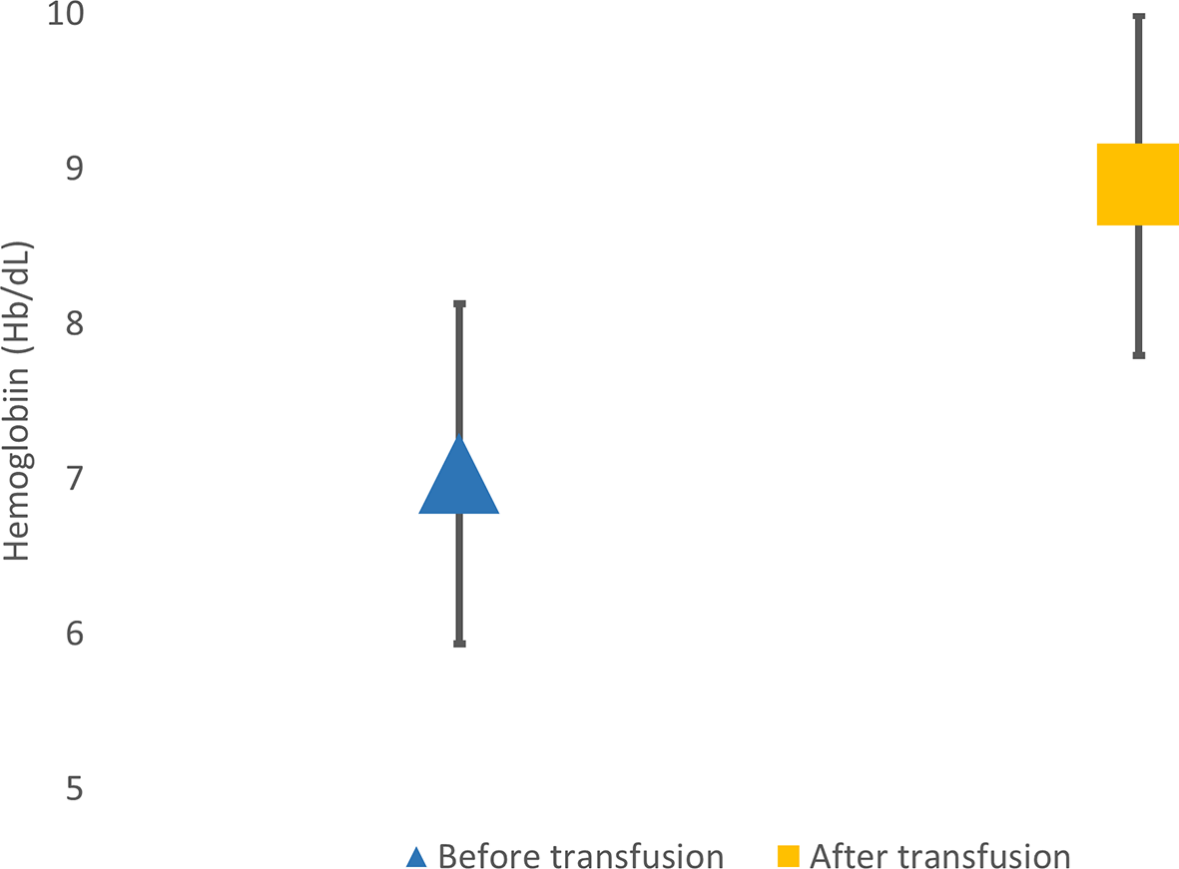
Hemoglobin level before and after transfusion.

Most of the subjects have never been examined for their pain, so the cause of pain were not further assssesed. Based on the data of the study, the average pain intensity was moderate pain and disappeared after transfusion. The data can be seen in Figure 3.

Questions in the BPI-SF questionnaire about the treatment taken by the patient and the pain treatment effect were not included in the scoring assessment. This study showed that the most pain is reduced by transfusion in 20 (30%) patients with an effect of 40–100% pain reduction. Other treatments taken by the subjects are analgetics drugs, rest, compress, and iron chelation. There is an increase in the average hemoglobin before transfusion from 7±1.1 g/dL to 8.9±1.1 g/dL.

Pain intensity before transfusion had a median value of 5 while pain intensity after transfusion had a median value of 0. These numerical data were analyzed using the Wilcoxon test. The analysis results showed a significant difference in pain intensity before and after transfusion with p=0.0001 (<0.05). A comparison of pain interfering with life obtained from BPI-SF scores before and after transfusion using the Wilcoxon test obtained p=0.0001 (<0.05); accordingly, there is the significant difference in pain interfering with life before and after transfusion as listed in Table 2.

**Table 2.** Comparison of pain intensity and pain interfering with life using BPI-SF questionnaire before and after transfusion.

Variable	Group		P-Value
	Before transfusion	After transfusion	
	N=60	N=60	
Pain intensity			0.0001 **
Median	5	0	
Range (min-max)	1.8-7.5	0-7	
Pain interferes with life			0.0001 **
Median	2.8	0	
Range (min-max)	0-8.1	0-7.4	

** p<0.05 was considered significant.

## Discussion

Most of the subjects did not disclose their pain to the doctor when they went to the Haemato-Oncology Clinic. There were only two subjects who checked for pain and carried out supporting examinations to determine the pain cause. Forty-nine other subjects were known to have pain based on the results of filling out the BPI-SF questionnaire. Low hemoglobin in thalassemia causes hypoxia and releases reactive oxygen species (ROS) through inhibition activity of oxygen-sensitive prolyl hydroxylases (PHDs), then causes the activation of transient receptor potential ankyrin 1 (TRPA1) and transient receptor potential vanilloid 1 (TRPV1). ^10^^,^^11^ This protein activation will release substance P which plays the role in pain sensitization. ^12^

TRPA1, a nonselective cation channel, is widely expressed in nociceptive C fibers, dorsal root ganglia (DRG) and trigeminal ganglia neurons. ^11^ Human nerve fibers are susceptible to free radicals such as ROS since it is high in phospholipids and mitochondria; furthermore, the neurons antioxidant defenses are weak. TRPV1 is a nonselective cation channel expressed mainly in unmyelinated C nerve fibers detecting and integrating pain stimuli. TRPV1 can be sensitized by exposure to hypoxia and can induce pain. ^10^ The role of TRPV1 and TRPA1 in thalassemia-related pain are thought to be associated with arthritis. These two molecules can be activated in sensory neurons, chondrocytes and synoviocytes. ^20^

Pain in thalassemia is associated with musculoskeletal involvement with a wide variety of symptoms. ^21^ The most common complaints of musculoskeletal pain are arthritis and low back pain. ^1^^,^^22^^,^^23^ The musculoskeletal pain primary location is in the lower back affecting 70-85% of the adult population. ^24^ Pain-sensitive structures in the lower back are involved in pain such as the periosteum of the bones, ligaments, facets, articular capsule, and paraspinal muscles. ^25^^–^^27^ The lower back anatomy has characteristics making this area more prone to pain than the other parts of the back. The foramina in the lumbar vertebrae are small, triangular, and narrow at the lateral angle at the L4-L5 vertebrae. The nerve roots are located in this lateral recess before exiting the intervertebral foramen more prone to compression. Each vertebra adjusts its shape and size as the reflection of the load it receives. The lumbar vertebrae has larger size than the other vertebrae, the load supported by the lower back is greater than the rest of the back and has more significant impact in trauma. The load received by the spine varies depending on the posture and external loads. The L3-L4 intervertebral discs in the sitting position receive higher load than when standing, the pressure is at the lowest when lying supine. ^26^

The most common affected location in this study is the lower back in 22 (43.1%) patients. Low back pain is caused by osteophytes, facet hypertrophy, fractures and osteoporosis causing pain in the bones. Low back pain is also triggered by prolonged standing and heavy lifting and is mainly due to low hemoglobin which will improve after transfusion. ^28^^,^^29^

The bone mass rapid turnover results from an imbalance between increased bone resorption and suppression of osteoclasts. This process is more common in the lumbar spine as a result of extramedullary erythropoiesis. The hemoglobin level before transfusion was 8.5-9 g/dL, it may be clinically tolerated by adults with thalassemia; nonetheless, the process of extramedullary erythropoiesis can continue to occur. It is recommended that the hemoglobin before transfusion be maintained at above 10 g/dL level to prevent extramedullary erythropoiesis in these patients' thalassemia. Decreased bone mass in thalassemia often occurs in the vertebral column and can manifest as spinal deformity, bone marrow compression, vertebral collapse, and intervertebral disc degeneration. ^30^^–^^32^ Matrix metalloproteinases can underlie the arthritis occurrence. ^33^ Pain in the lower back can also be caused by low bone density causing fractures and compression in the spinal cord. ^4^ The most common finding in the vertebrae MRI examination is the degenerative vertebral disc with the highest prediction for L4-L5 intervertebral discs. ^34^

The second most common location is the knee while the rest complain the pain in more than one location in the body such as in the joints of the fingers and toes, calves, ankles, ankles, heels and shoulders. This condition is also known as thalassemic osteoarthropathy. ^35^

There is a role for Hypoxia Inducible Factor (HIF) in arthritis. HIF acts as the regulator of the adaptive response to hypoxia; hence, HIF is involved in the inflammation persistence and neovascular progression in arthritis. HIF has several target genes including erythropoietin (EPO) supporting the erythropoiesis. HIF is released under hypoxic conditions, yet it can also be released under normoxia by several inflammatory factors such as ROS, nitric oxide and proinflammatory cytokines such as IL-1β and TNF alpha. ^11^^,^^21^

HIF-1α hydroxylation is inhibited and accumulates in the cytoplasm under hypoxic conditions. This causes HIF-1α to be phosphorylated and translocated to the nucleus, in which it binds to the HIF-1β subunit to form a complex [HIF-α/HIF-1b]. This complex via HRE (Hypoxia Release Element) binds to a specific DNA sequence (5′TAGCGTGH3′) in the promoter region of several genes such as including EPO (erythropoietin). HIF-2α is the cartilage catabolism regulator working as a matrix-degrading enzyme and inflammation mediator (IL-1, IL-6, and TNF-α), increasing the expression of proteolytic enzymes and MMPs (Matrix Metalloproteinases), accelerating cartilage destruction and causing chondrocyte hypertrophy, all of which play the role in the arthritis development. ^36^

This study showed that the subjects experienced improvement in the life quality after transfusion. Judging from the results of filling out the BPI-SF questionnaire by adults with TDT, 94.12% of the subjects experienced pain decrease after transfusion. It is found in Table 2 that there are significant differences between pain intensity and pain interfering with life obtained from BPI-SF scores before and after transfusion. This study is in line with the previous studies that revealed pain in thalassemia is commonly associated with low hemoglobin at the end of the transfusion cycle relieved by transfusion. ^1^

### Strengths and limitations

This study emphasizes the importance of carrying out pain assessments for thalassemia patients. This study is the first to validate the BPI-SF tools into the Indonesian version and this questionnaire can be used in other studies as well related to chronic pain.

The following limitations are not further considered in this study. The examination was carried out to determine the pain etiology according to the fact that the subjects did not disclose their pain to the doctor when they went to the Hemato-Oncology Clinic. Most of the subjects knew their pain diagnose based on the results of filling out the BPI-SF questionnaire.

Further studies are needed to determine the pain etiology in adults with TDT, especially those related to low back pain and arthritis as the most common pain causes in adults with TDT using the cross-sectional approach.

## Conclusion

The conclusion of this study is that the pain can occur in thalassemia patients due to low hemoglobin level and can be relieved with transfusion. There is the significant difference between pain intensity and pain interfering with life before and after transfusion.

## Data availability

F1000 Research: Data set 1. The data for BPI-SF validation per item question. https://doi.org/10.6084/m9.figshare.16984870

Data set 2. Demographic data of adult TDT patients. https://doi.org/10.6084/m9.figshare.17032340

Data set 3. The data of BPI-SF score and the hemoglobin level of adult TDT patients before and after transfusion. https://doi.org/10.6084/m9.figshare.17032361
