# Supplementary material for: A Comparison of Pain Before and After Transfusion in Adult transfusion-dependent thalassemia (TDT) Using BPI-SF
Source: F1000Res. 2024 Aug 9;12:1425. Originally published 2023 Oct 31. [Version 2] doi: 10.12688/f1000research.75952.2 (PMC11421607; doi:10.12688/f1000research.75952.2)
Supplement: Peer review history

# Reviewer report: Reviewer response for version 2

**DOI:** 10.5256/f1000research.170012.r317035

**Published:** 2024-09-20

**Authors:** Donna Wall

**Affiliations:** 1 University of Toronto, Toronto, Canada

The main focus of this manuscript is exploring the use of the Brief Pain Inventory - short form in the setting of a referral program that provides care for patients with transfusion-dependent thalassemia.  

 The group first validated a version of the BPI-SF that had been translated into Indonesian and applied to a test group.  They then studied 60 consecutive patients prior to and post transfusion of a single unit of red cells (hemoglobin avg went from 7 to 8.9 g) and repeat questionnaire was performed within 24 h of transfusion.  

 The major observation was that pain was a common problem and not reported by the patients at their visits - perhaps because it is such a pervasive/chronic issue.  The response to even a modest change in hemoglobin was striking and in followup it would be interesting to understand the duration of improvement.  The cohort had a low pre-transfusion hemoglobin level for a chronically transfused cohort - this raises the question of whether the degree of improvement would also be seen in a cohort where pre-transfusion hemoglobins were higher.

 While the discussion of pathophysiology of pain in patients with thalassemia is interesting, the focus of the paper is the development/application of the BPI-SF to clinical care.  Does the research team have recommendations on how this patient reporting tool could/should be integrated into the clinical practice - perhaps helping with targeting transfusion frequency or targets for trough hemoglobin?  A brief exploration of applicability to practice would strengthen the impact of the results.  

 Including patient reporting is an under appreciated care tool and this study has demonstrated the ability to apply it to a practice in a thalassemia clinic in Indonesia.

Is the work clearly and accurately presented and does it cite the current literature?

Yes

If applicable, is the statistical analysis and its interpretation appropriate?

Yes

Are all the source data underlying the results available to ensure full reproducibility?

Yes

Is the study design appropriate and is the work technically sound?

Yes

Are the conclusions drawn adequately supported by the results?

Yes

Are sufficient details of methods and analysis provided to allow replication by others?

Yes

Reviewer Expertise:

hematology and bone marrow transplant with a focus on hemoglobinopathies

I confirm that I have read this submission and believe that I have an appropriate level of expertise to confirm that it is of an acceptable scientific standard.

# Reviewer report: Reviewer response for version 2

**DOI:** 10.5256/f1000research.170012.r312730

**Published:** 2024-09-09

**Authors:** Noor Syahireen Mohammed

**Affiliations:** 1 Clinical Research Centre, Sultanah Bahiyah Hospital, Alor Setar, Kedah, Malaysia

No further comments

Is the work clearly and accurately presented and does it cite the current literature?

Partly

If applicable, is the statistical analysis and its interpretation appropriate?

Partly

Are all the source data underlying the results available to ensure full reproducibility?

Yes

Is the study design appropriate and is the work technically sound?

Partly

Are the conclusions drawn adequately supported by the results?

Partly

Are sufficient details of methods and analysis provided to allow replication by others?

Yes

Reviewer Expertise:

Health system policy

I confirm that I have read this submission and believe that I have an appropriate level of expertise to confirm that it is of an acceptable scientific standard.

# Reviewer report: Reviewer response for version 1

**DOI:** 10.5256/f1000research.79898.r232558

**Published:** 2024-02-06

**Authors:** Noor Syahireen Mohammed

**Affiliations:** 1 Clinical Research Centre, Sultanah Bahiyah Hospital, Alor Setar, Kedah, Malaysia

The study provides a significant contribution to the understanding of pain experienced by thalassemia patients. The research's focus, methodology, significant results, and plausible findings are noteworthy. However, the study has certain limitations that restrict the generalizability of the findings. These limitations must be addressed as this would enhance the study's conclusions and help in developing better pain management strategies for thalassemia patients.

 In detail, some of the strong points are:

 •    Clearly defined research question: The study investigates a relevant issue – pain management in adult TDT patients – with a specific and focused question about the impact of blood transfusion.

 •    Appropriate sound methodology: The study utilizes valid inclusion and exclusion criteria, an appropriate sample size for paired data analysis, and established pain assessment tools with proper validity and reliability testing.

 •    Statistically significant results: The observed decrease in pain intensity and interference following transfusion is statistically significant, supporting the main hypothesis.

 •    Plausible explanation: The discussion provides a scientific explanation for the findings, linking pain reduction to improved haemoglobin levels and reduced activation of pain receptors.

 •    Well-written and informative: The study is clearly presented and easy to understand, using appropriate language and structure.

 However, some improvements can be made on these points or addressed by author:

 Introduction:

 •    Lacks a clear opening statement to capture the reader's attention and set the stage for the research.

 •    Could benefit from a stronger connection between the background information and the research question.

 •    Mentions only one potential cause of pain (low haemoglobin) without acknowledging other possible contributors.

 •    Doesn't explicitly state the hypothesis or main argument of the study.

 Methodology:

 •    Limited information on sampling technique (random vs. convenience etc.)

 •    Potential for bias in data collection due to self-reporting through telephone interviews.

 •    Limited information on pain assessment methods beyond BPI-SF 

 •    Lack of consideration for potential confounding variables (e.g., medication use, psychological factors).

 Results:

 •    Limited generalizability due to potential bias and reliance on self-reported data.

 •    No analysis of other potential causes of pain besides low haemoglobin.

 Discussion:

 •    Limited exploration of alternative explanations for pain beyond low haemoglobin and musculoskeletal involvement.

 •    Offers limited explanation for the potential variation in individual responses and treatment effectiveness.

 •    Doesn't discuss the limitations of pain assessment tools like BPI-SF in this context.

 In conclusion, this study provides very useful information on pain management for Thalassemia patients. However, the study has some limitations that need to be addressed, such as the sampling technique, the potential for bias, and a need for a more comprehensive investigation into pain mechanisms. Addressing these limitations would help strengthen the conclusions and lead to more generalizability of the methodology to help with policymaking.

Is the work clearly and accurately presented and does it cite the current literature?

Partly

If applicable, is the statistical analysis and its interpretation appropriate?

Partly

Are all the source data underlying the results available to ensure full reproducibility?

Yes

Is the study design appropriate and is the work technically sound?

Partly

Are the conclusions drawn adequately supported by the results?

Partly

Are sufficient details of methods and analysis provided to allow replication by others?

Yes

Reviewer Expertise:

Health system policy

I confirm that I have read this submission and believe that I have an appropriate level of expertise to confirm that it is of an acceptable scientific standard, however I have significant reservations, as outlined above.

# Response

**Published:** 2024-08-04

**Authors:** Uni Gamayani

**Affiliations:** Perdossi, Indonesia

Thank you for your review. I have revised my article and I sent it as version 2 article.

# Reviewer report: Reviewer response for version 1

**DOI:** 10.5256/f1000research.79898.r232559

**Published:** 2024-01-08

**Authors:** Xuemei Zhen

**Affiliations:** 1 Shandong University, Jinan, Shandong, China

**Introduction** **:**

1. Need to describe what is known about the issue based on current literature.
2. Mention your study innovation.
3. Need to state what the gap in the literature is and how this study helps to fill the gap in knowledge.
4. Why choose adult patients? State what problems adult TDT patients have that impose more pain.
5. The fourth paragraph should be mentioned in the Method section.
6. The purpose of the study needs to be modified. The objective is not clearly stated or specific.

**Method:**

1. Please describe each instrument separately in the data collection section.
2. The sample size formula should be mentioned and the calculation process should be explained to ensure that the sample size was sufficient for this study.
3. What variables and data were collected?
4. Validity and reliability of the instrument should be mentioned. If you used the Indonesian version of the instruments, please mention the references.
5. Please describe each instrument more specifically and in detail.
6. Mention the year of design of the BPI-SF questionnaire.
7. Explain better why it was not possible to include a larger sample.
8. In the statistical analysis, you mentioned “the data was obtained by telephone interview”. How were the interviews conducted? Is there a risk of bias in this situation?
9. Please provide evidence of applying any measures of quality control such as "cherries survey checklist" or any other publicly available quality control measures.

**Result:**

1. In the first paragraph, the presentation of the analytical methods and tools used should be contained in the Method section.
2. In the second paragraph, “the duration of being diagnosed with thalassemia” needs to be mentioned in Table 1, as well as “other treatments taken”.
3. Describe the educational level and employment status of the sample.

**Discussion: **

1. First paragraph should include the most important findings of the study.
2. In the third and fourth paragraph, the portion describing the lower back pain should be grouped into one paragraph.
3. Please explain more about the comparison with the results of other studies rather than describing the principles that lead to pain.
4. The discussion has not adequately compared the current study with other new studies. Many new studies have been conducted in the field of thalassemia patients in different countries. While the authors have used old references.
5. Various variables have been examined in the study, but these variables have not been examined in the discussion. For example, the different demographic variables.

**Strengths and limitations**

1. Sample size should be one of the limitations.

**Conclusion**

1. Mention the application of your findings in the fields of education, research, and clinical practice.

**References**

1. The references are very old and not acceptable at all. At least 50% of the references should be from the last 5 years.

Is the work clearly and accurately presented and does it cite the current literature?

Partly

If applicable, is the statistical analysis and its interpretation appropriate?

Partly

Are all the source data underlying the results available to ensure full reproducibility?

Partly

Is the study design appropriate and is the work technically sound?

Partly

Are the conclusions drawn adequately supported by the results?

Partly

Are sufficient details of methods and analysis provided to allow replication by others?

Partly

Reviewer Expertise:

My area of research is social medicine, health economic and policy.

I confirm that I have read this submission and believe that I have an appropriate level of expertise to confirm that it is of an acceptable scientific standard, however I have significant reservations, as outlined above.
